# Beyond fixed beats: adaptive musical entrainment for precision neurorehabilitation in neurodegenerative disorders

**DOI:** 10.3389/fresc.2026.1909811

**Published:** 2026-09-23

**Authors:** Benoît G. Bardy

**Affiliations:** EuroMov Digital Health in Motion, University of Montpellier, IMT Mines Alès, Montpellier, France

**Keywords:** 1/f variability, adaptive music, auditory-motor coupling, digital therapeutics, music-based neurorehabilitation, Parkinson's disease, phase synchronization, rhythmic auditory stimulation

## Abstract

Music-based interventions have become a promising avenue for neurorehabilitation in neurodegenerative disorders, particularly through rhythmic auditory stimulation to improve gait and movement timing. However, many current interventions still rely on pre-specified or externally imposed rhythmic structures, treating music primarily as a cue to be followed. In this Opinion article, I argue that the next generation of music-based neurorehabilitation should move beyond fixed beats or individualized cueing protocols toward real-time adaptive musical entrainment. Two biologically grounded principles are central to this shift. First, healthy movement is not metronomic: gait exhibits structured variability, including long-range correlations and 1/f-like temporal organization, which can be disrupted in neurodegenerative disease. Because music can contain ‘pink,’ multiscale temporal structure in specific acoustic or rhythmic time series, it offers a promising medium for adaptive cue design and for testing whether structured auditory variability can support more functional movement organization. Second, adaptive music should be understood not merely as tempo matching, but as adaptive auditory-motor timing control in which phase synchronization is one important dimension, among others. Closed-loop musical technologies can continuously sense movement, adapt musical parameters, and support stable yet flexible coupling between body and sound. Because the empirical foundation for music-based gait rehabilitation is currently strongest in Parkinson's disease, this article uses PD gait as the primary model while treating extension to atypical parkinsonian syndromes and other neurodegenerative disorders as a translational hypothesis requiring disease-specific validation. I propose that precision neurorehabilitation may integrate individual movement signatures, structured temporal variability, and phase-based adaptive cueing to guide the development of interventions that are more personalized, engaging, and ecologically grounded. Music, in this framework, does not simply tell the patient when to move; it listens back and adapts to support more functional movement dynamics.

## 1 Music-based neurorehabilitation: promise and limits of feedforward cueing

Music-based interventions are among the clearest examples of how external stimulation can support motor rehabilitation in neurodegenerative disorders. In Parkinson's disease, rhythmic auditory stimulation (RAS) has shown that external rhythm can improve gait timing, cadence, stride length, and walking stability (1–3). Meta-analyses and clinical studies indicate positive effects of rhythmic cueing on gait, while also emphasizing heterogeneity in response and the need for better-powered, ecologically valid trials (4–6). Music adds further value because it engages auditory, motor, cognitive, affective, and motivational systems simultaneously (3, 7).

However, rhythm-based interventions should not be reduced to the mechanical alignment of steps with beats. For RAS to influence gait, several distinguishable processes may be involved: auditory beat perception, beat-based prediction, neural entrainment, perceptual-motor synchronization, and the eventual behavioral gait response. Here, neural entrainment refers to rhythm-related modulation or alignment of neural activity to an external auditory rhythm. It should be distinguished from predictive motor engagement, which refers to the recruitment of motor timing and prediction processes during beat perception, and from behavioral synchronization, which refers to the observable alignment between movement events and musical events. Recent work suggests that beat perception engages motor and predictive processes even in the absence of overt movement, supporting the idea that the motor system participates in the construction of temporal expectations (8). Neuroimaging evidence further indicates that musical rhythm recruits a bilateral cortico-subcortical network including auditory cortex, supplementary motor area, putamen, and cerebellum (9). A recent scoping review of auditory-motor entrainment similarly emphasizes the role of supplementary motor area, premotor cortex, basal ganglia, and cerebellum in auditory-motor timing (10). During walking, these processes must operate while the patient turns, avoids obstacles, divides attention, adapts to fatigue, and responds to environmental and emotional changes. Such considerations raise the hypothesis that responsiveness to RAS may differ across parkinsonian disorders, depending not only on gait impairment but also on disease-specific differences in beat perception, prediction, synchronization capacity, and rhythm-processing networks.

RAS has evolved considerably beyond simple metronomic stimulation. Existing approaches include individualized tempo selection, progressive cueing protocols, musical rather than purely metronomic stimuli, assessment of rhythmic abilities, and patient-specific recommendations (e.g. (11). These developments have been essential for translating auditory cueing into clinical and home-based rehabilitation. Conventional open-loop RAS is also not non-interactive in an absolute sense: the patient actively perceives the rhythm, predicts its timing, senses the beat-step relation, and adjusts movement accordingly. The key distinction is that, in open-loop RAS, the patient's movement changes the perceived beat-step relation but does not change the auditory stimulus itself. The shift proposed here is therefore not from stimulus to interaction in a strict sense, but from pre-specified or individualized feedforward cueing toward real-time, movement-contingent coupling in which movement also informs how the musical stimulus evolves.

This limitation matters because neurodegenerative disorders alter more than average movement parameters. They affect variability, adaptability, fatigue, attention, and the capacity to maintain coordination under changing constraints. A patient walking at home must turn, stop, avoid obstacles, divide attention, and respond to changing internal states. Precision neurorehabilitation therefore requires more than a fixed external clock. It requires systems that can detect the current movement state, adapt musical structure accordingly, and guide movement without eliminating its natural flexibility. I propose that music-based neurorehabilitation should move beyond fixed beats toward adaptive musical entrainment, grounded in two complementary principles: structured temporal variability as a therapeutically relevant property of gait dynamics, and adaptive auditory-motor timing control in a coupled body-music system.

In the sections that follow, I distinguish between established findings, theoretical interpretation, technological feasibility, and future research hypotheses. The evidence that RAS can improve gait is strongest in Parkinson's disease. The evidence that gait contains long-range temporal correlations, and that cueing structure can influence these correlations, supports the relevance of temporal complexity but does not establish 1/f scaling as a validated therapeutic target, standalone biomarker, or global measure of locomotor health. Similarly, phase variables have already been operationalized as mechanistic and control variables in adaptive cueing, but their status as clinical endpoints or standalone digital biomarkers requires further validation. The framework proposed here should therefore be read as a roadmap for precision music-based rehabilitation rather than as a claim that these mechanisms have already been fully validated across neurodegenerative disorders.

## 2 Healthy movement is not metronomic: 1/f structure and the loss of temporal complexity

A central limitation of the fixed-beat paradigm is that it can confuse health with regularity. Healthy gait is not a perfect clock. Even during steady walking, stride intervals fluctuate from one step to the next, and these fluctuations are not random noise around an ideal rhythm. They show long-range correlations and fractal-like temporal organization (12). This finding changed how movement variability is interpreted. Too much variability may signal instability, but too little variability may signal rigidity. Healthy biological systems often operate between these extremes, maintaining structured variability that supports adaptation across multiple time scales (13). However, there is no universal optimum independent of the variability measure, temporal scale, task, and clinical population. The goal is therefore not to define a single ideal alpha-DFA range for all patients, but to understand when structured variability reflects adaptive gait organization in a given context.

Neurodegenerative disorders can disrupt this temporal organization. Work on gait dynamics has shown that aging, Huntington's disease, and Parkinson's disease can alter stride-to-stride correlations and fractal-like scaling, with changes that are not reducible to mean speed or stride length (14, 15). From this perspective, one candidate therapeutic goal is to preserve, and potentially restore, functional variability: a form of temporal organization that remains stable enough to support walking and flexible enough to adapt.

Music is especially relevant because it is not merely periodic. Many musical signals contain 1/f-like, or ‘pink,’ fluctuations in loudness, rhythm, pitch, and expressive timing (16–18). In the present context, ‘pink’ does not refer to music as a whole, but to 1/f-like temporal structure in specified time series, such as fluctuations in acoustic energy, rhythmic event timing, inter-beat intervals, or expressive timing. This multiscale structure may help explain why music feels predictable yet alive. A metronome provides a beat; music provides pulse plus variation. The distinction is clinically important. If healthy gait contains structured temporal dynamics, and if neurodegenerative disease can degrade those dynamics, then music may provide a temporal environment that supports more functional movement organization. Hove et al. (19) showed that interactive RAS can influence 1/f-like gait timing in Parkinson's patients under tested conditions, whereas fixed-tempo stimulation did not show the same effect. We have more recently shown that rhythmic cues embedding biological variability preserve long-range correlations in gait in both Parkinson's patients and healthy controls, whereas isochronous or randomly variable cueing removes this structure when participants synchronized strongly with the cues (20). These findings support structured temporal variability as a promising target for adaptive cue design, not as a standalone clinical endpoint. A technical limitation is that alpha-DFA estimates are methodologically sensitive: values obtained from short or heterogeneous walking series may not be directly comparable with estimates from longer controlled sequences. Changes in alpha-DFA should therefore be interpreted cautiously, as process-level effects of cue–movement coupling, and not necessarily as individual-level evidence of restored locomotor health (21, 22).

## 3 Adaptive music as timing control: from fixed cueing to adaptive auditory-motor coupling

The second limitation of fixed cueing is not that it lacks auditory-motor entrainment, but that the evolving state of entrainment is not itself used to regulate subsequent cueing. In conventional RAS, the patient adjusts movement relative to an externally prescribed or individualized auditory reference. In a closed-loop adaptive system, the evolving movement state and movement-music relationship can additionally be estimated and used to modify the auditory stimulus in real time. In this sense, the novelty is not auditory-motor entrainment itself but making the evolving state of entrainment part of the control loop. A functional phase relation may need to be individualized according to baseline walking, synchronization capacity, comfort, safety constraints, and therapeutic goals such as guiding cadence, reducing asymmetry, stabilizing gait, or preventing loss of coupling.

This distinction is central to adaptive musical entrainment. Human movement unfolds through oscillatory processes: stepping, swaying, reaching, breathing, and interpersonal coordination all involve rhythmic components that can couple to external rhythms. Music also unfolds as a temporal structure organized around pulse, meter, accent, and expectation. Theories of dynamic attending describe rhythmic perception as the entrainment of internal attentional oscillations to external events (23), while sensorimotor synchronization research emphasizes phase and period correction as distinct processes (24). At a broader dynamical level, Kuramoto-like models show how oscillators with different natural frequencies can become phase-locked when coupling is appropriately tuned (25). In this paper, the Kuramoto-like framing is used as a conceptual analogy for coupled timing dynamics, not as evidence that clinical controllers are mathematically equivalent to Kuramoto systems or should implement a single phase-locking rule. Phase synchronization is therefore one dimension of auditory-motor coordination, not its complete explanation.

Applied to neurorehabilitation, this means that adaptive music should not merely estimate cadence and impose a corrected tempo. It should estimate movement state, adapt musical timing, and support a clinically meaningful timing relation between steps and sound. In BeatWalk/BeatMove-type systems (e.g. (26, 27), gait phase is estimated from detected footfalls using wearable sensors, the musical reference is the annotated beat or pulse event in the music, and the controller can operate at the step level, while extensions to full gait cycles or separate left-right coordination remain possible depending on the therapeutic objective. Phase error refers to the instantaneous offset between a step event and a musical reference event; period change refers to a sustained change in the step-to-step interval; and intentional cadence change refers to a therapist- or protocol-defined target shift in walking rhythm. Coupling strength, phase lag, phase variability, and recovery after perturbation are established mechanistic and control variables in adaptive cueing, but their status as clinical endpoints or standalone digital biomarkers requires validation against functional outcomes such as gait speed, endurance, freezing, falls, participation, and quality of life. The controller should also avoid reinforcing pathological patterns: if excessive asymmetry, festination, freezing-like behavior, abrupt cadence changes, or uncertainty in phase estimation is detected, adaptation should be reduced, simplified, suspended, or returned to a therapist-defined scaffold. Our own multiscale account of beat-based movement emphasizes that auditory-motor coupling spans physical, behavioral, and neural levels (28), that interaction and predictability shape spontaneous entrainment (29), and therefore that adaptive music should be conceived as a responsive partner in a perception-action loop (Figure 1). rather than a non-responsive or externally prescribed temporal scaffold.

**Figure 1:**
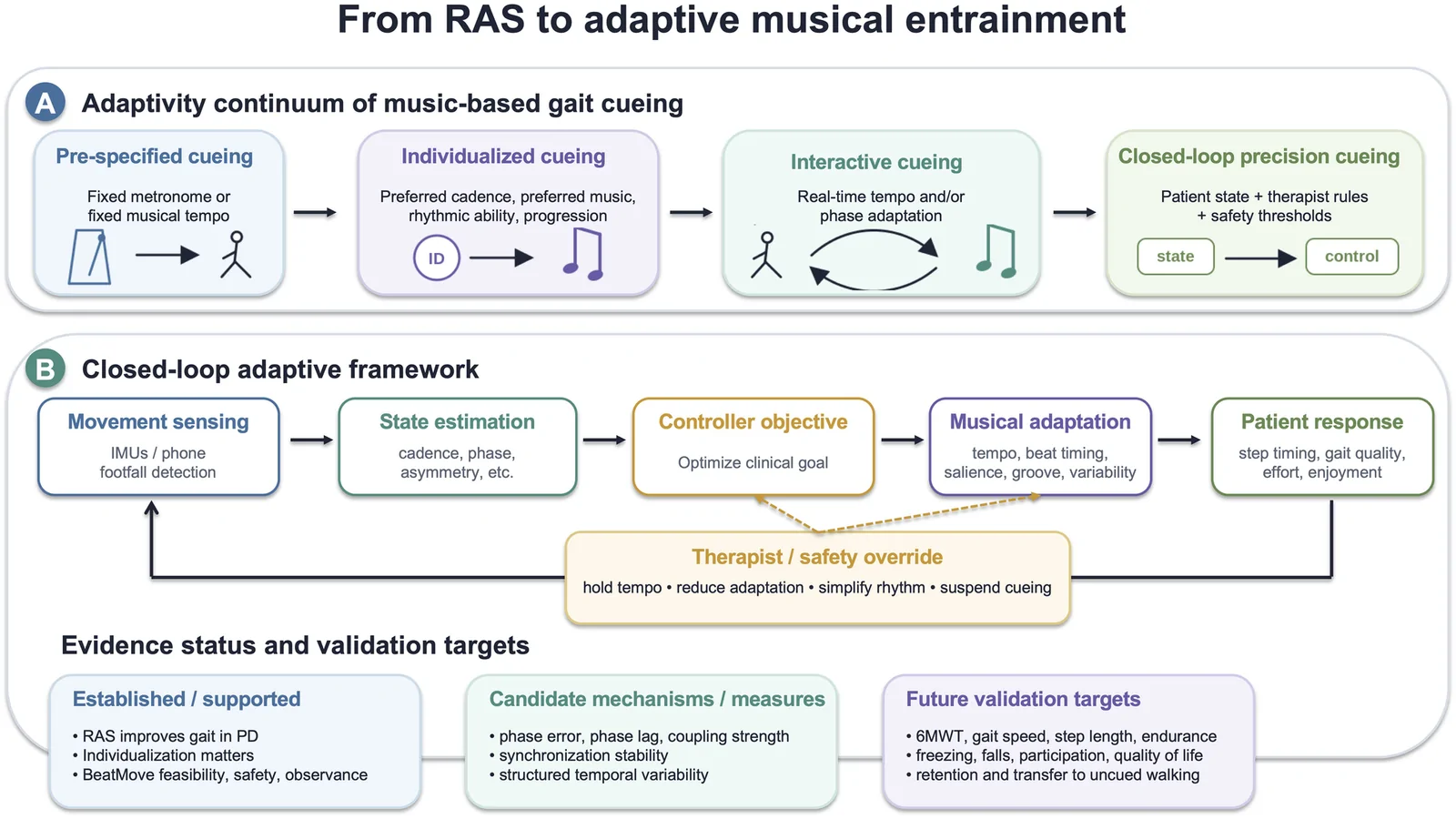
Adaptivity continuum of music-based gait cueing and closed-loop adaptive framework. Panel **(A)** presents music-based gait cueing as a continuum ranging from pre-specified rhythmic stimulation to individualized, interactive, and fully movement-contingent closed-loop approaches. Conventional RAS already involves active auditory-motor regulation by the patient; the proposed extension is that movement also informs how the musical cue evolves. Panel **(B)** separates sensing, state estimation, controller objective, musical adaptation, patient response, and therapist/safety override. In this framework, wearable sensors estimate movement state, including cadence, gait phase, asymmetry, and variability. A controller then adapts musical parameters according to a clinically defined objective, which may involve maximizing synchronization, maintaining synchronization within an individualized range, guiding cadence, supporting gait quality, preserving safety, reducing effort, or sustaining engagement. Phase-related variables and structured temporal variability are presented as mechanistic and control variables or candidate process measures, not as standalone validated clinical endpoints. Future studies should test whether these measures predict clinically meaningful outcomes such as gait speed, endurance, freezing, falls, participation, quality of life, retention, and transfer to uncued real-world walking.

## 4 Toward precision musical entrainment: individual movement signatures and adaptive control

The move toward adaptive musical entrainment also changes the meaning of precision. In many rehabilitation settings, personalization still means adjusting tempo to the patient's preferred cadence or setting a target slightly above baseline. This is useful, but limited. Patients with the same diagnosis may differ in rhythmic ability, gait variability, fatigue profile, responsiveness to cueing, emotional engagement, and capacity to maintain synchronization. These differences help explain why the same rhythmic intervention can improve gait in one patient and have little effect, or even destabilizing effects, in another (30, 31).

Precision musical entrainment should also account for possible differences across parkinsonian disorders. RAS is most often discussed in Parkinson's disease, but atypical parkinsonian syndromes may not share the same auditory-neural-motor pathway for using external rhythm. Recent fMRI evidence comparing auditory beat perception in Parkinson's disease and multiple system atrophy reported different disease-related activation patterns, including greater cerebellar activation in Parkinson's disease and greater premotor/precentral activation in multiple system atrophy (32). These findings motivate hypotheses about disease-specific rhythm-processing networks, but they do not by themselves demonstrate differences in gait synchronization or clinical response to RAS, nor do they necessarily prove compensation. Greater activation may reflect compensation, effort, inefficiency, dysfunction, or alternative strategy. Before adaptive music can optimize movement timing, the system must assume that patients may differ in how they perceive beats, generate internal timing, and translate auditory rhythm into motor coordination. Cognitive impairment, cerebellar involvement, freezing of gait, postural instability, medication state, and disease severity may all affect feasibility, safety, and responsiveness. Disease-specific rhythm-processing profiles may therefore become an important component of precision neurorehabilitation, but this remains a hypothesis requiring direct validation.

A precision approach should therefore be based on individual movement signatures. These include conventional clinical variables such as cadence, speed, stride length, asymmetry, and endurance, but also candidate mechanistic and control variables such as stride-to-stride correlations, phase locking, phase variability, coupling strength, synchronization stability, and recovery after perturbation. This view is consistent with the proposal to individualize music-based rhythmic cueing in Parkinson's disease (11) and with the broader need to integrate digital mobility outcomes into clinically meaningful assessment (33). The key point is not that every derived variable is already a validated clinical biomarker, but that adaptive cueing creates candidate digital measures that can be tested against conventional and patient-centered outcomes.

A central challenge for adaptive musical entrainment is determining the direction and degree of adaptation. Pathological gait is not always cleanly oscillatory: phase estimates may be ambiguous, step timing may be irregular, and asymmetry between the two legs may complicate the definition of a single gait phase. Adaptive systems should therefore not be conceived as algorithms that simply follow the patient's current cadence. Rather, they should operate as clinically constrained controllers. When gait phase is reliable, music can adapt gradually to support a desired phase relation. When phase estimation is uncertain, the appropriate action may be to hold tempo, reduce the strength of adaptation, simplify the rhythmic structure, or return to a therapist-defined scaffold. In asymmetric gait, adaptation may require separate left–right phase estimates, coupling to the more stable limb, or targeting the global gait cycle rather than individual steps. Thus, the question is not whether adaptive music should always accelerate, slow, or stabilize, but under which clinical and dynamical conditions each action is appropriate. Properly designed, adaptive RAS can preserve the stabilizing function of an external temporal scaffold while adding the ability to detect loss of coupling and adjust only when doing so is safe and meaningful.

Closed-loop technologies make this approach increasingly feasible. Wearable sensors and smartphones can capture movement continuously, while adaptive algorithms can modify musical parameters in response to ongoing gait dynamics (34). Obviously, the technical capacity to sense movement continuously should not be equated with validated continuous clinical assessment; clinically meaningful interpretation of these measures still requires validation against established functional outcomes. Adaptive and closed-loop cueing are not specific to the BeatMove program. Hove et al. (19) provided an independent interactive-RAS precedent, using real-time coupling between gait and auditory rhythm to influence 1/f-like gait timing in Parkinson's disease. Although outside the neurodegenerative scope of the present article, InTandem provides a recent independent example of autonomous closed-loop auditory-motor entrainment in chronic stroke, using real-time gait sensing to adapt music tempo and beat salience while gating progression through gait-quality criteria (35). Recent autonomous or synchronized music-based cueing studies in Parkinson's disease also support the ecological relevance of this direction (36, 37). Within this broader field, the BeatMove research program contributes specific evidence on personalized music-based gait rehabilitation in Parkinson's disease, including feasibility, safety, enjoyment, observance, and real-time beat-step coupling (26, 27, 38). Unlike InTandem, whose published description emphasizes cadence-to-tempo alignment, tempo progression, beat salience, symmetry, and variability, the BeatMove framework places stronger emphasis on beat-step relative phase and phase-based musical coupling. Independent replication and comparative trials remain necessary to determine which components are most effective. The scientific point, however, is broader than any single system: music-based rehabilitation can become reciprocal, such that the patient shapes the music and the music shapes movement. Such reciprocity can generate candidate digital measures, including synchronization stability, preferred phase relation, adaptation rate, and responsiveness to musical structure, whose clinical value remains to be established.

## 5 From intervention to adaptive coupling: future directions for music-based neurorehabilitation

Adaptive musical entrainment reframes music-based rehabilitation along an adaptivity continuum rather than as a simple contrast between stimulus and interaction. In pre-specified cueing, auditory structure is selected in advance; in individualized cueing, tempo, musical style, or progression are adapted to the patient before or between sessions; in interactive cueing, the patient continuously perceives, predicts, and adjusts movement relative to the rhythm; and in fully movement-contingent systems, sensing and control rules allow music to adapt online to the evolving movement state. Conventional RAS therefore already involves perception-action regulation on the patient side. The additional contribution of closed-loop adaptation is to change the directionality and adaptability of the coupling, allowing body and music to mutually constrain each other within clinically defined safety limits (Figure 1).

Several testable questions follow. First, does real-time movement-contingent adaptation improve synchronization stability beyond pre-specified or individualized cueing? Second, do biologically structured or 1/f-informed cue structures preserve or modulate stride-to-stride organization more effectively than isochronous or randomly variable cues? Third, do candidate coupling measures such as phase variability, coupling strength, adaptation rate, and recovery after loss of synchronization predict clinical outcomes such as gait speed, step length, endurance, freezing, falls, participation, quality of life, retention, and transfer to uncued walking? Fourth, which musical attributes–beat clarity, groove, familiarity, affective tone, preference, and 1/f-like temporal organization–drive auditory-motor coupling, enjoyment, and adherence? These features may covary in natural music, and experimental designs will be needed to isolate their respective contributions.

Future trials should also include comparator conditions capable of separating the effects of real-time adaptation from related but distinct components. Relevant comparators include walking without cueing, preferred music without rhythmic adaptation, fixed-tempo or tempo-personalized music, beat-salient music without real-time adaptation, delayed or sham adaptation, and fully adaptive movement-contingent music. Such designs are necessary to distinguish the effects of physical activity, music preference, beat salience, expectancy, simple tempo personalization, and real-time movement-contingent adaptation.

Finally, adaptive systems must remain clinically transparent and safe. If movement is sensed continuously, privacy, data governance, and interpretability are central. If algorithms adapt music in real time, clinicians and patients should understand the principles guiding adaptation, including algorithmic accountability, user autonomy, accessibility, and adverse-event monitoring. Safety criteria should include uncertainty thresholds for state and phase estimates, fall-risk monitoring, freezing or festination detection, excessive asymmetry or instability, cognitive-load considerations, medication-state awareness, therapist oversight, and explicit criteria for holding tempo, reducing adaptation strength, simplifying rhythmic structure, or suspending cueing. The question is no longer whether rhythm can influence movement. The next question is how rhythm, music, body, and technology can be coupled to support functional movement in ways that are individualized, engaging, safe, and sustainable. Music, in this framework, does not simply tell the patient when to move; it listens back and adapts to support more functional movement dynamics.
